# Preparation of Silicophosphate Alternating Hybrid Copolymers via Nonaqueous Acid-Base Reactions of Phosphoric Acid and Organo-Bridged Bis(chlorosilane)

**DOI:** 10.3390/molecules25010127

**Published:** 2019-12-28

**Authors:** Kenji Okada, Masanari Takano, Yasuaki Tokudome, Yomei Tokuda, Masahide Takahashi

**Affiliations:** 1 Department of Materials Science, Graduate School of Engineering, Osaka Prefecture University, Sakai, Osaka 599–8531, Japan; 2 JST, PRESTO, 4-1-8 Honcho, Kawaguchi, Saitama 332-0012, Japan; 3 Faculty of Education, Shiga University, Otsu, Shiga 520-0862, Japan

**Keywords:** silicophosphate, nonaqueous acid–base reaction, organo-bridged silane, organic-inorganic hybrid material, cage molecule, phosphoric acid, density functional theory, alternating copolymer.

## Abstract

A design of atomic and oligomer level structure in organic-inorganic hybrid materials is highly important for various applications. Nonaqueous acid-base reaction allows us to prepare silicophosphates with controlled inorganic networks (–(O–P–O–Si)_n_) at atomic level because phosphorous and silicon-based precursors can react directly, resulting in an alternating copolymer network. Organic functionalization in those materials has been realized so far by using organic-modified phosphorous acid and/or organo-chlorosilane as precursors. In the present study, silicophosphate oligomers exhibiting inorganic-organic hybrid chains of (–(O–P–O–Si–R–Si)_n_) (R: bridging organic functional groups), are prepared from phosphoric acid and organo-bridged bis(chlorosilane). The 1, 2-bis(chlorodimethylsilyl)ethane ((C_2_H_4_)(Me_2_SiCl)_2_) and 1, 4-bis(chlorodimethylsilyl)benzene ((C_6_H_4_)(Me_2_SiCl)_2_) were used as organo-bridged bis(chlorosilane). Different types of silicophosphate oligomers with different network structures and terminal groups (P-OH and/or Si-Cl) were prepared by changing the reaction temperature and molar ratio of precursors. The formation of low molecular weight oligomers of ring and cage morphologies (ring tetramer, cage pentamer, and ring hexamer) is suggested in the product prepared from phosphoric acid and (C_6_H_4_)(Me_2_SiCl)_2_ molecule at 150 °C. Those silicophosphate hybrid oligomers are expected to be used as building blocks of hybrid materials with well-defined network structures for desired functionalities.

## 1. Introduction

Organic-inorganic hybrid materials have attracted attention because of the unique properties derived from both organic and inorganic moieties. As one of the famous hybrid materials, polydimethylsiloxane (PDMS) is widely used for applications ranging from medical applications to optical devices due to its optical transparency, non-toxicity, non-flammability, and mechanical flexibility. The properties of the hybrid materials are mostly determined by the chemistry of the organic and inorganic components, their arrangements, and the microstructures [1,2]. These hybrid materials are widely synthesized via an alkoxide-derived sol-gel method because the low temperature process prevents the decomposition of the organic component [3,4,5]. However, the conformation of the organic and inorganic components is hardly controllable by the alkoxide-derived sol-gel method because the hydrolysis and condensation reactions of inorganic moieties of organically modified alkoxides take place competitively, which results in different nucleation-growth processes. A solvent-free sol-gel method which involves the nonhydrolytic condensation and nonaqueous acid-base reaction allowed us to prepare highly controlled inorganic networks at an atomic level because precursors can react directly with the counterpart [6,7,8]. We have reported silicate-phosphate (silicophosphate) and silicate-phosphite (silico-phosphite) alternating copolymers (–(O–P–O–Si)_n_) by a direct reaction of phosphoric acid (or phosphorous acid) and organically modified chlorosilane [9,10,11,12]. The network formation process involved a nonaqueous acid-base reaction as follows: P–OH + Si–Cl → P–O–Si + HCl↑. Thus, the phosphorous and silicon precursors are alternatingly linked by oxo bridges. The resultant hybrid materials exhibited low glass transition temperatures around at −25 °C to 70 °C, which allowed for the homogeneous introduction of optically active organic dyes and rare-earth ions in the hybrid materials for optical applications [13]. It was found that the molecular structures and the oligomer-scale networks were related to the chemical durability and the thermal properties of the hybrid materials [14,15]. Silicophosphate oxo bridges (Si-O-P) were quite hygroscopic due to the hydrolysis by water in ambient atmosphere. Usually, size and hydrophobicity of the organic group largely affect the stability. It has been reported that the phenyl-modified silicophosphate materials with 100% degree of condensation exhibit good stability in an ambient atmosphere. This was achieved by introducing bulky and hydrophobic moiety like phenyl group and also by the highly linked well-developed oxo networks [14]. On the other hand, it is well known that the network dimension and degree of condensation largely affect the thermal property of the resultant polymeric materials, especially for the softening temperature. Actually, it is expected for the organically modified silicophosphate materials as one of alternatives of so-called “low-melting glasses” which have softening (glass transition) temperature around 100 °C–400 °C. We have also reported that the silicophosphate materials are one of the candidates for such alternative materials [15,16]. Thus, the design of these structures is important to control the chemical, thermal, and optical properties.

In the present study, silicophosphate hybrid oligomers, which contain organic moieties in inorganic main chains, were prepared from phosphoric acid and organo-bridged bis(chlorosilane) by nonaqueous acid-base reactions. The 1,2-bis(chlorodimethylsilyl)ethane ((C_2_H_4_)(Me_2_SiCl)_2_) and 1,4-bis(chlorodimethylsilyl)benzene ((C_6_H_4_)(Me_2_SiCl)_2_) were used as organo-bridged bis(chlorosilane). In the silicophosphates reported so far, organic functional groups were introduced as the side chain of the (–(O–P–O–Si)_n_) networks. The use of organo-bridged bis(chlorosilane) as precursors resulted in the incorporation of organic moieties in the inorganic networks as (–(O–P–O–Si–R–Si)_n_) (R: Bridging organic functional groups), which attain the further flexibility for a design of atomic level structures in the hybrid materials. In addition, the formation of regular structures, such as layered structures and cage structures, is expected in the hybrid materials as reported in the conventional sol-gel derived organo-silica, metal organic framework, and metal-organic polyhedra, which provide us sophisticated properties, such as gas sorption, and unique optical and mechanical properties [17,18,19,20,21]. Herein, different types of silicophosphate oligomers were prepared from phosphoric acid and organo-bridged bis(chlorosilane) by simply changing the reaction temperature and molar ratio of precursors. The structures of silicophosphate oligomers were investigated by nuclear magnetic resonance (NMR) and gel permeation chromatography (GPC). The silicophosphate oligomers are expected to be used as building blocks for preparing hybrid materials with well-defined molecular structures for desired functionalities.

## 2. Results and Discussion

### 2.1. Reactivity between Phosphoric Acid and Organo-Bridged Bis(Chlorosilane) and the Preparation of Silicophosphate Oligomers

**Figure 1:**
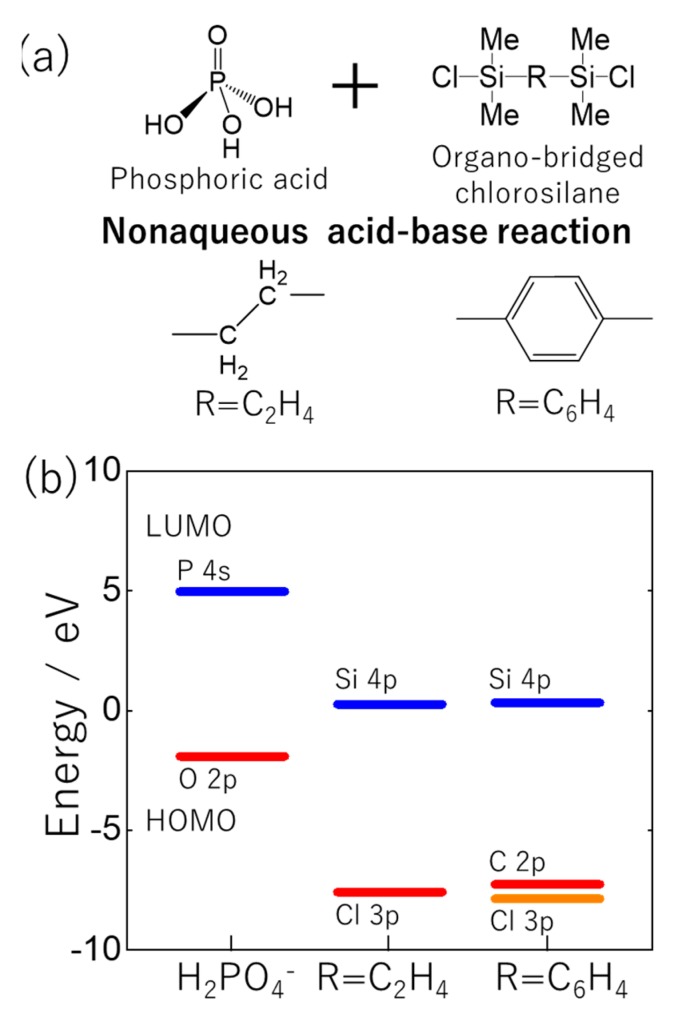
(**a**) Precursors used in the present study, (**b**) HOMO and LUMO energy levels of H_2_PO_4_^−^, (C_2_H_4_)(Me_2_SiCl)_2_ and (C_6_H_4_)(Me_2_SiCl)_2_ molecules evaluated by DFT calculation.

The silicophosphate hybrid oligomers were synthesized by a nonaqueous acid-base reaction between phosphoric acid and organo-bridged bis(chlorosilane) ((C_2_H_4_)(Me_2_SiCl)_2_ or (C_6_H_4_)(Me_2_SiCl)_2_) (Figure 1). In this method, the precursors were mixed under N_2_ atmosphere and then gradually heated to predetermined temperatures. Then, the solutions were kept at the temperature for 5.5 h and cooled to an ambient temperature. In the nonaqueous conditions, H_2_PO_4_^−^ anions were produced by autoprotolysis of phosphoric acid. The H_2_PO_4_^−^ anion as nucleophilic reagent attached to silicon in organo-bridged bis(chlorosilane) by S_N_2(Si) addition reaction, resulting in the formation of P-O-Si linkage. According to the reaction mechanism, the energy gap between HOMO energy level of H_2_PO_4_^−^ anion and LUMO energy level of organo-bridged bis(chlorosilane) determined the reactivity between phosphorus and silicon precursors and the yield of the resultant hybrid materials^12^. Figure 1b shows the HOMO and LUMO energy levels of the precursors. The HOMO level of the H_2_PO_4_^−^ anion was contributed by the O-2p orbital. LUMO levels of the organo-bridged bis(chlorosilane) were constructed mainly by the Si-4p orbital. The LUMO levels with the same energy were confirmed in the (C_2_H_4_)(Me_2_SiCl)_2_ and (C_6_H_4_)(Me_2_SiCl)_2_ precursors, indicating that the both silicon precursors exhibited the same reactivity to phosphoric acid.

### 2.2. Silicophosphate Hybrid Network Formation from Phosphoric Acid and (C_2_H_4_)(Me_2_SiCl)_2_

**Figure 2:**
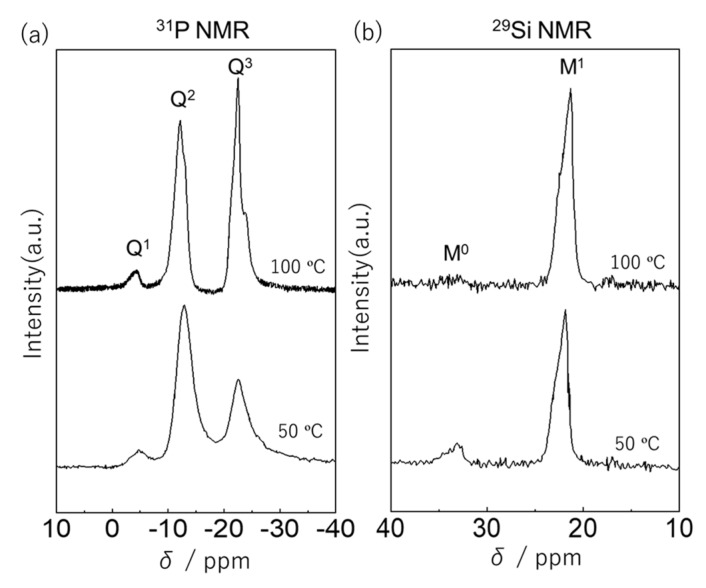
(**a**) The ^31^P and (**b**) ^29^Si NMR spectra of the products prepared from phosphoric acid and (C_2_H_4_)(Me_2_SiCl)_2_ at 50 °C and 100 °C.

The phosphoric acid and (C_2_H_4_)(Me_2_SiCl)_2_ were mixed with a stoichiometric composition (H_3_PO_4_: (C_2_H_4_)(Me_2_SiCl)_2_ =1:1.5) and reacted in THF at 50, 100, and 150 °C. Spinnable and transparent products were obtained. Figure 2 shows ^31^P and ^29^Si NMR spectra of the products prepared at 50 and 100 °C. In the ^31^P NMR spectra, three distinct peaks were observed at −4.8, −12.8, and −22.5 ppm, which were assigned to Q^1^, Q^2^, and Q^3^ units, respectively. The fraction of each unit was calculated from the NMR spectra. The higher reaction temperature of 100 °C led to a decrease of the fraction of Q^2^ unit from 66.5% for 50 °C to 46.5% for 100 °C and an increase of that of Q^3^ unit from 29.2% to 50.0% with maintaining that of Q^1^ unit (4.3% for 50 °C and 3.5% for 100 °C). This result indicates that both products contained P-OH at the terminal of the (–(O–P–O–Si–C_2_H_4_–Si)_n_) networks and the products prepared at 100 °C had more branched networks. In the ^29^Si NMR spectra, two distinct peaks were observed at 33.1 and 21.6 ppm, which were assigned to M^0^ and M^1^ units. The fractions of M^0^ and M^1^ units for the product prepared at 50 °C were calculated as 14.8% and 85.2%. In the product prepared at 100 °C, M^1^ unit was the dominant structural unit as the fractions of M^0^ and M^1^ units were 0.05% and 99.5%, respectively. A similar result was observed for that at 150 °C (Figure S1). GPC investigation indicated that the resultant products were constituted of lower molecular weight molecules than 1000 g/mol, which was calculated according to the molecular weight of standard polystyrene. From these results, it was concluded that the product prepared at 50 °C contained both P-OH and Si-Cl at the terminal of the networks, while the terminal of the networks at 100 °C and 150 °C was mostly P-OH. The formation of linear and/or cyclic oligomers was assumed in the product prepared at 50 °C by comparing with those at 100 °C and 150 °C because the linear Q^2^ unit is the main structural unit. Cyclic oligomers and polyhedral (e.g., cage and double-decker) likely form in the products prepared at 100 °C and 150 °C because the branched Q^3^ unit was the main structural unit and the fraction of Q^1^ unit was relatively low compared to that of the Q^3^ unit.

### 2.3. Silicophosphate Hybrid Network Formation from Phosphoric Acid and (C_6_H_4_)(Me_2_SiCl)_2_

**Figure 3:**
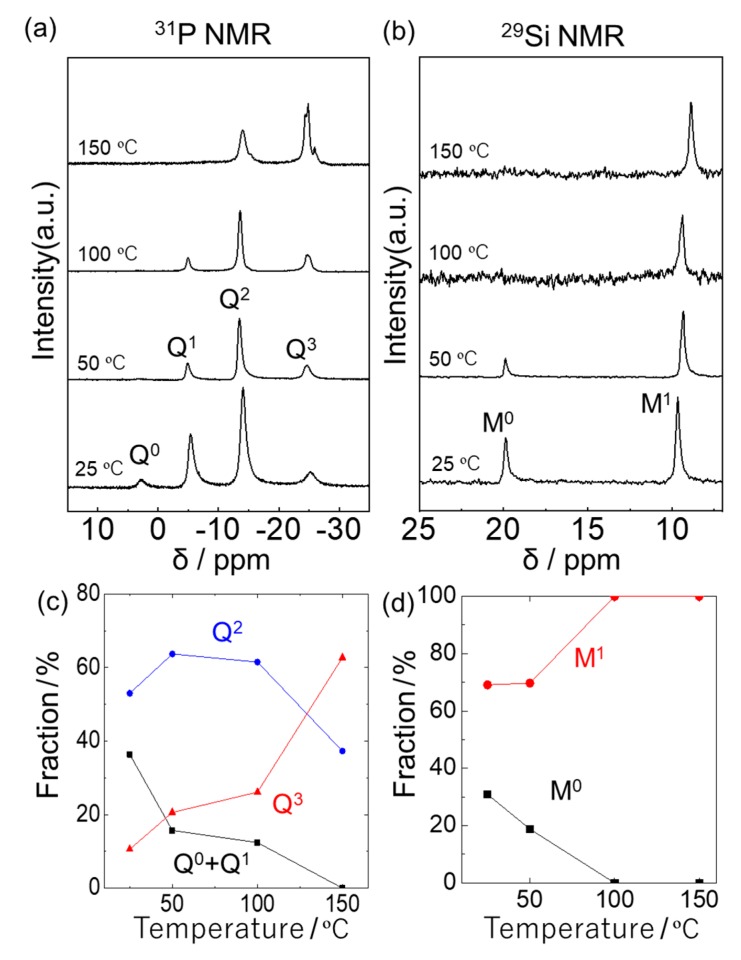
(**a**) The ^31^P and (**b**) ^29^Si NMR spectra of the products prepared from phosphoric acid and (C_6_H_4_)(Me_2_SiCl)_2_ at each temperature. (**c**) The fraction of Q^0^, Q^1^, Q^2^, and Q^3^ units at each temperature calculated from ^31^P NMR spectra. (**d**) The fraction of M^0^ and M^1^ units at each temperature calculated from ^29^Si NMR spectra.

Clear and viscous products with a bit brownish color were obtained by a reaction between phosphoric acid and (C_6_H_4_)(Me_2_SiCl)_2_ in THF with a stoichiometric composition (H_3_PO_4_: (C_6_H_4_)(Me_2_SiCl)_2_ = 1:1.5) at 25, 50, 100, and 150 °C. Figure 3a,b shows ^31^P and ^29^Si NMR spectra of the products prepared at each temperature. The peaks at 2.8, −5.1, −13.5, and −24.8 ppm in the ^31^P NMR spectra are assigned to Q^0^, Q^1^, Q^2^, and Q^3^ units, respectively. The peaks at 19.9 and 9.2 ppm in the ^29^Si NMR spectra are assigned to M^0^ and M^1^ units. Figure 3c,d shows the fraction of each unit at different reaction temperatures. The phosphoric acid exhibited higher reactivity to (C_6_H_4_)(Me_2_SiCl)_2_ molecules at higher temperatures. The ^29^Si NMR investigation showed that all chlorine in (C_6_H_4_)(Me_2_SiCl)_2_ molecules reacted with the phosphoric acid, resulting in the formation of P-O-Si linkage at the reaction temperature over 100 °C. Notably, the formation of only Q^2^ and Q^3^ structural units was observed in the product prepared at 150 °C, indicating that the product contained no terminal P-OH and Si-Cl in the networks and phosphoric acid, and (C_6_H_4_)(Me_2_SiCl)_2_ molecules were alternatingly connected to each other. Under this condition, the fractions of Q^2^ and Q^3^ units were 37.3% and 62.7%, respectively. The result indicates the formation of cyclic oligomers as well as cage molecules at 150 °C.

**Figure 4:**
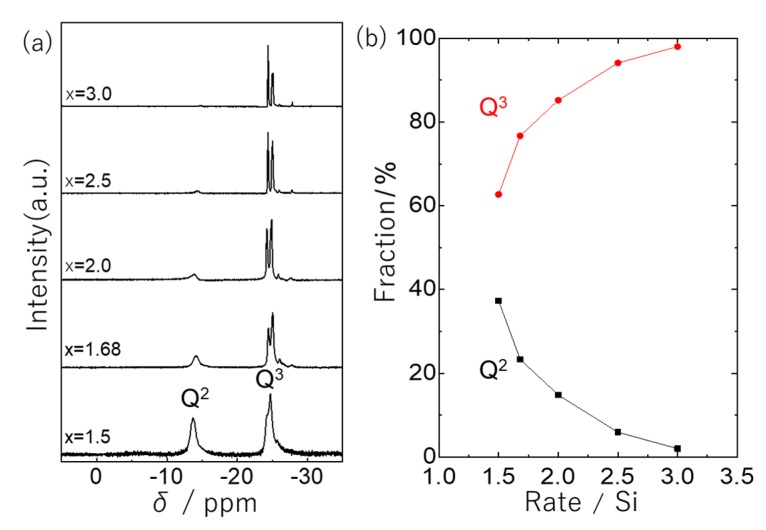
(**a**) The ^31^P NMR spectra and (**b**) the corresponding fraction of Q^2^ and Q^3^ units at each composition calculated from ^31^P NMR spectra in the H_3_PO_4_-(C_6_H_4_)(Me_2_SiCl)_2_ system.

The effect of composition, in terms of molar ratio, of H_3_PO_4_: (C_6_H_4_)(Me_2_SiCl)_2_ =1:x (x = 0.5, 1.0, 1.5, 1.68, 2.0, 2.5, 3.0) was investigated at 150 °C reaction temperature. When the molar ratio of silicon precursor was less than stoichiometric composition (x < 1.5), viscous products, that were insoluble in tetrahydrofuran (THF), were obtained (therefore, NMR measurement was not conducted for these products). This is presumably because high-molecular weight silicophosphate is prepared by a self-condensation of phosphoric acid. Figure 4 shows ^31^P NMR spectra and the corresponding fraction of the products prepared at different compositions (x = 1.5, 1.68, 2.0, 2.5, 3.0). An increase of Q^3^ unit and a split of the peak related to Q^3^ unit were observed with increasing the molar fraction of the (C_6_H_4_)(Me_2_SiCl)_2_. The peak splitting indicates the formation of Q^3^ unit in different chemical environments. At x = 3.0, the network was composed of mostly branched Q^3^ units linked by linear (C_6_H_4_)(Me_2_SiCl)_2_ molecules. In that product, the terminal group of the inorganic networks was determined as Si-Cl because both M^0^ and M^1^ units were confirmed by ^29^Si NMR investigation (Figure S2). The products prepared at x = 1.5 and 3.0 were investigated by GPC (Figures S3–S6). The product of x = 1.5 was constituted of only lower molecular weight molecules, like cyclic and cage molecules less than 1000 g/mol, which was calculated according to the standard polystyrene. On the other hand, the composition at x = 3.0 resulted in the silicophosphate alternating copolymers with a wide range of molecular weight from 200 to 10,000 g/mol.

In the present study, different types of silicophosphate oligomers with different network structures (such as cyclic and cage oligomers) and terminal groups (P-OH and/or Si-Cl) were prepared by changing the reaction temperature and molar ratio of the precursors. Although various types of molecular silicophosphates, such as cyclic and cage oligomers, have been reported so far [22,23,24,25,26], this is the first report on the synthesis of silicophosphates oligomers which contain organic moieties in inorganic main chains. The organic moieties incorporated in the inorganic chains of silicophosphates can increase the surface area as reported in silicophosphate xerogels prepared from bridged acetoxysilanes and phosphoryl reagents [27]. This advantage allows for the further use as building blocks for preparing hybrid materials with well-defined network structures for desired functionalities.

## 3. Materials and Methods

Orthophosphoric acid, H_3_PO_4_ (≥99.999%, Sigma-Aldrich Co., Tokyo, Japan), 1, 2-Bis(Chlorodimethylsilyl)ethane, (C_2_H_4_)(Me_2_SiCl)_2_ (96%, Sigma-Aldrich Co.), and 1, 4-Bis(Chlorodimethylsilyl)benzene, (C_6_H_4_)(Me_2_SiCl)_2_ (95%, Sigma-Aldrich Co.) were used as precursors. All the samples were prepared under a N_2_ atmosphere as reported [9]. For the H_3_PO_4_-(C_2_H_4_)(Me_2_SiCl)_2_ system, H_3_PO_4_ and (C_2_H_4_)(Me_2_SiCl)_2_ were dissolved in 9 mL of tetrahydrofuran (THF) with a molar ratio of H_3_PO_4_: (C_2_H_4_)(Me_2_SiCl)_2_ = 1:1.5 with stirring and then gradually heated to 50, 100, and 150 °C in 30 min. Then, the solutions were kept at each temperature for 5.5 h and cooled to ambient temperature. The H_3_PO_4_-(C_6_H_4_)(Me_2_SiCl)_2_ system was conducted by the same procedure as the H_3_PO_4_-(C_2_H_4_)(Me_2_SiCl)_2_ system. The compositions of the precursors in this system were changed as H_3_PO_4_: (C_6_H_4_)(Me_2_SiCl)_2_ = 1:x (x = 0.5, 1.0, 1.5, 1.68, 2.0, 2.5, 3.0).

The ^31^P NMR and ^29^Si NMR spectra were measured using Varian Unity-Inova (300 MHz) and JEOL ECX400, ECS400. The samples for NMR measurements were prepared by 3-fold diluting the resulting viscous liquid with chloroform-d (98.00%, Wako Pure Chemical Industries, Ltd., Tokyo, Japan) and THF (super dehydrated with stabilizer, ≥99.5%, Wako Pure Chemical Industries, Ltd.). The chemical shift of ^31^P was obtained relative to that of 85% H_3_PO_4_. The chemical shift of ^29^Si NMR was obtained relative to that of tetramethyl silane.

The molecular weights of the samples were estimated by a GPC with Tosoh CO-8020, RI-8020, DP-8020, and SD-8022 systems (Tosoh Co., Tokyo, Japan) equipped with a guard column (Tosoh TSKgel guard column SuperHZ-L, Tosoh Co., Tokyo, Japan) and three analytical columns (two Tosoh TSKgel SuperHZ1000 columns, and a TSKgel SuperHZ2000 column (Tosoh Co., Tokyo, Japan)) at 40 °C. Polystyrene standards (TSKgel Standard Polystyrene, Tosoh Co., Tokyo, Japan) were employed for the calibration. Tetrahydrofuran (THF)was used as the eluent at a flow rate of 1.0 mL/min.

The DFT calculations at the B3LYP/6-31+G(d,p) level were carried out for the H_2_PO_4_^−^, (C_2_H_4_)(Me_2_SiCl)_2_, and (C_6_H_4_)(Me_2_SiCl)_2_ using the Gaussian 09 program [28]. The DFT calculations were also performed for the cage-type (PO)_2_(Ph(Me_2_SiO)_2_)_3_ molecules to determine the stabilized molecular structure.

## 4. Conclusions

Silicophosphate oligomers with alternating hybrid copolymer structures were prepared from phosphoric acid and organo-bridged bis(chlorosilane) ((C_2_H_4_)(Me_2_SiCl)_2_ or (C_6_H_4_)(Me_2_SiCl)_2_) by nonaqueous acid-base reactions. In the H_3_PO_4_-(C_2_H_4_)(Me_2_SiCl)_2_ system with a stoichiometric composition, P-OH or Si-Cl terminated linear and/or cyclic oligomers were mainly prepared at 50 °C and P-OH terminated cyclic oligomers and polyhedral (e.g., cage and double-decker) oligomers were likely obtained at 100 and 150 °C. In the H_3_PO_4_-(C_6_H_4_)(Me_2_SiCl)_2_ system, at the stoichiometric composition, P-OH or Si-Cl terminated oligomers were obtained at temperature below 100 °C. Importantly, low molecular weight ring and cage oligomers (ring tetramer, cage pentamer, and ring hexamer) were considered to be synthesized in the product prepared at 150 °C. At the 150 °C reaction temperature, an excess of phosphoric acid (x = 0.5 and 1.0) led to a self-condensation of phosphoric acid that impeded the formation of P-O-Si linkages, while the excess of (C_6_H_4_)(Me_2_SiCl)_2_ resulted in the Si-Cl terminated products with a wide range of molecular weight where almost all phosphorous had three P-O-Si linkages with (C_6_H_4_)(Me_2_SiCl)_2_ molecules.

The present synthetic approach for the alternating hybrid copolymers opens the simple and facile way to prepare small rings or cages that are expected for applications in gas sorption, drug delivery, and others as micro structure building units with a small cavity within them.

## Supplementary Materials

The following are available online at https://www.mdpi.com/1420-3049/25/1/127/s1, Figure S1: The ^1^P and ^29^Si NMR spectra and GPC data of the product prepared from phosphoric acid and (C_2_H_4_)(Me_2_SiCl)_2_ at 150 °C. Figure S2: the ^29^Si NMR spectra of the products prepared from phosphoric acid and (C_6_H_4_)(Me_2_SiCl)_2_ at x = 1.5 and 3.0. Figure S3: GPC data for the product prepared at x = 1.5 and 3.0. Figure S4: the ^1^P NMR spectrum and GPC data for the product prepared from phosphoric acid and Me_2_PhSiCl at 100 °C. Figure S5: Possible molecular structures of a cage oligomer made from two phosphoric acid and three (C_6_H_4_)(Me_2_SiCl)_2_ molecules. Figure S6: Schematic illustration of the stabilized molecular structure of cage oligomer.
