# Supplementary material for: Preparation of Silicophosphate Alternating Hybrid Copolymers via Nonaqueous Acid-Base Reactions of Phosphoric Acid and Organo-Bridged Bis(chlorosilane)
Source: Molecules. 2019 Dec 28;25(1):127. doi: 10.3390/molecules25010127 (PMC6983256; doi:10.3390/molecules25010127)
Supplement: Supplementary file 1 [file molecules-25-00127-s001.pdf]

Supporting Information

Preparation of silicophosphate alternating hybrid copolymers via nonaqueous acid-base reactions of phosphoric acid and organo-bridged bis(chlorosilane)

Kenji Okada [1, 2,*], Masanari Takano [1], Yasuaki Tokudome [1], Yomei Tokuda [3], and Masahide Takahashi [1,*]

[1] Department of Materials Science, Graduate School of Engineering, Osaka Prefecture University, Sakai, Osaka, 599–8531, Japan

[2] JST, PRESTO, 4-1-8 Honcho, Kawaguchi, Saitama, 332-0012, Japan

[3] Faculty of Education, Shiga University, Otsu, Shiga, 520-0862, Japan

* Correspondence: okada@photomater.com (K.O.), masa@photomater.com (M.T.)

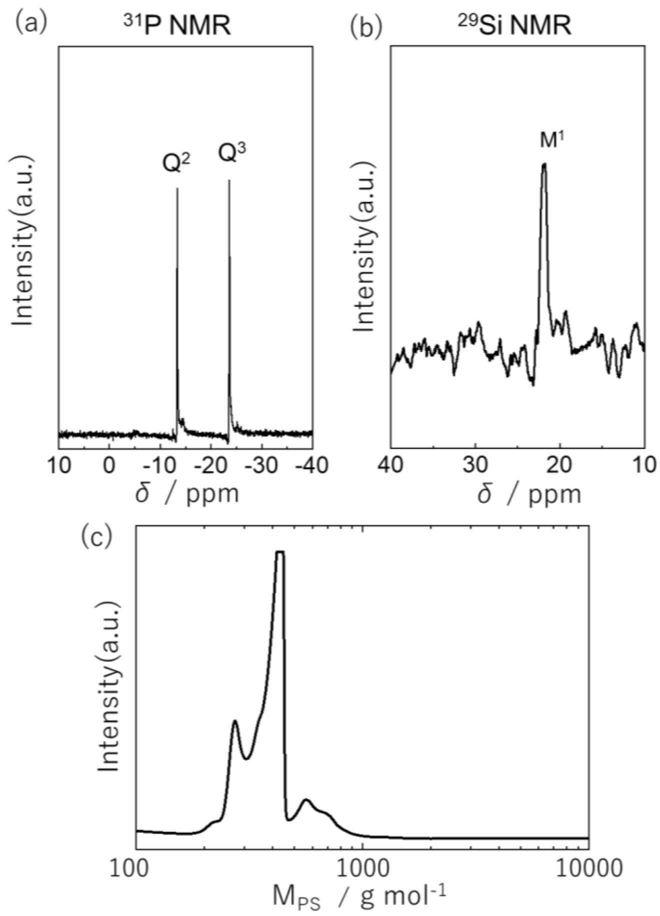

Figure S1. (a) The ^{31}P and (b) ^{29}Si NMR spectra of the product prepared from phosphoric acid and $(C_2H_4)(Me_2SiCl)_2$ at 150 ºC. (c) GPC data for the product. The molecular weight measured by GPC according to standard polystyrene is defined as M_{PS}

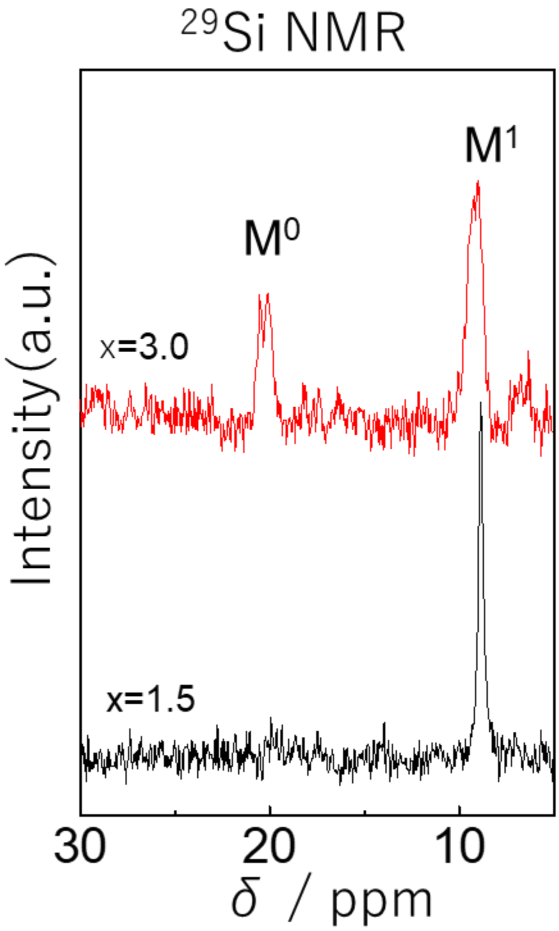

Figure S2. The ^{29}Si NMR spectra of the products prepared from phosphoric acid and $(C_6H_4)(Me_2SiCl)_2$ at x=1.5 and 3.0.

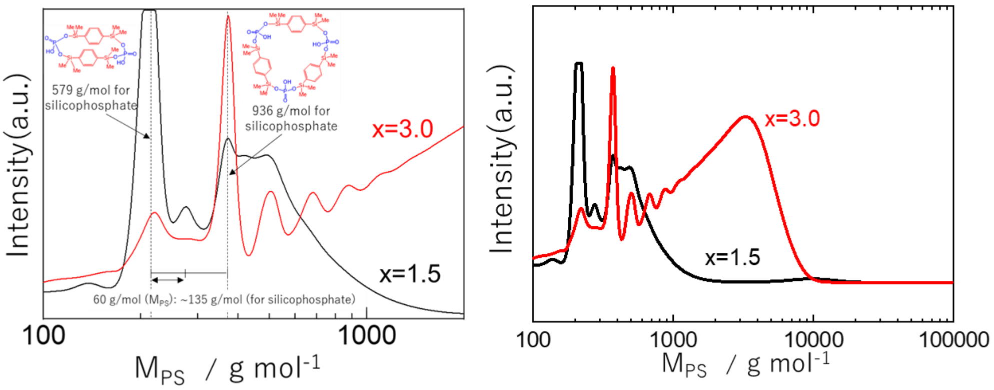

Figure S3. GPC data for the product prepared at x=1.5 and 3.0. The molecular weight measured by GPC according to standard polystyrene is defined as M_{PS}. The structures of the lower molecular weight molecules were investigated by a comparison with those of silicophosphate trimer and tetramer prepared as reference molecules (Figure S4). The above NMR investigation for the product prepared at x=1.5 showed no terminal P-OH and Si-Cl, indicating the formation of ring and cage oligomers. A smallest oligomer in this system is considered as a cyclic tetramer with a molecular weight of 579 g/mol made from two phosphoric acid and two $(C_6H_4)(Me_2SiCl)_2$ molecules. Thus, the silicophosphate with lowest molecular weight of M_{PS} 212 g/mol in GPC corresponds to the cyclic tetramer. The silicophosphate with M_{PS} 372 g/mol is considered to be ring hexamer made from three phosphoric acid and three $(C_6H_4)(Me_2SiCl)_2$ molecules because 936 g/mol estimated from a 160 g/mol M_{PS} difference from the cyclic tetramer and GPC data of reference silicophosphate molecules is close to the molecular weight of the ring hexamer (868 g/mol). The silicophosphate with M_{PS} 280 g/mol is estimated as a cage oligomer made from two phosphoric acid and three $(C_6H_4)(Me_2SiCl)_2$ molecules because the molecular weight of the cage oligomer is between those of the cyclic tetramer and the ring hexamer. These NMR and GPC investigations suggest the presence of cage oligomer made from two phosphoric acid and three $(C_6H_4)(Me_2SiCl)_2$ molecules in the product prepared at 150 °C and x=1.5. The possible cage oligomer structure is shown in Figure S6, of which the stabilized molecular structure is estimated from density functional theory (DFT) as shown in Figure S5.

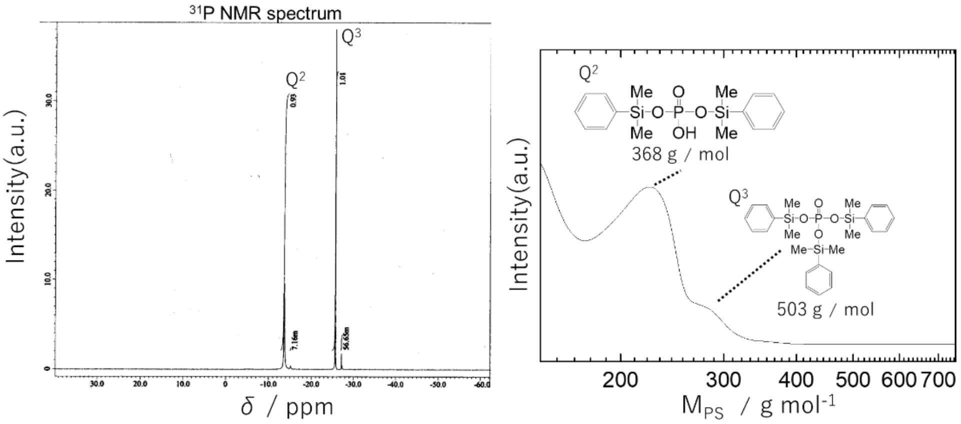

Figure S4. The ^{31}P NMR spectrum and GPC data for the silicophosphate trimer (OP(OH)-(Me_2PhSi-O)$_2$) and tetramer (OP-(Me_2PhSi-O)$_3$) prepared from phosphoric acid and $Me_2PhSiCl$. The silicophosphate trimer and tetramer were prepared from phosphoric acid and $Me_2PhSiCl$ at 100 °C with a stoichiometric composition (H_3PO_4: $Me_2PhSiCl$ =1:3). The ^{31}P NMR spectrum showed the presence of Q^2 (48%) and Q^3 unit (52%) without Q^1 unit, indicating the formation of both the silicophosphate trimer and tetramer. The GPC investigation for the trimer and tetramer showed that the M_{PS} of the trimer was 224 g/mol and that of tetramer was 284 g/mol. The calculated molecular weights of the trimer and tetramer are 366 and 501 g/mol, respectively. Thus, the 60 g/mol difference in M_{PS} is assumed to be 135 g/mol difference for silicophosphate oligomers.

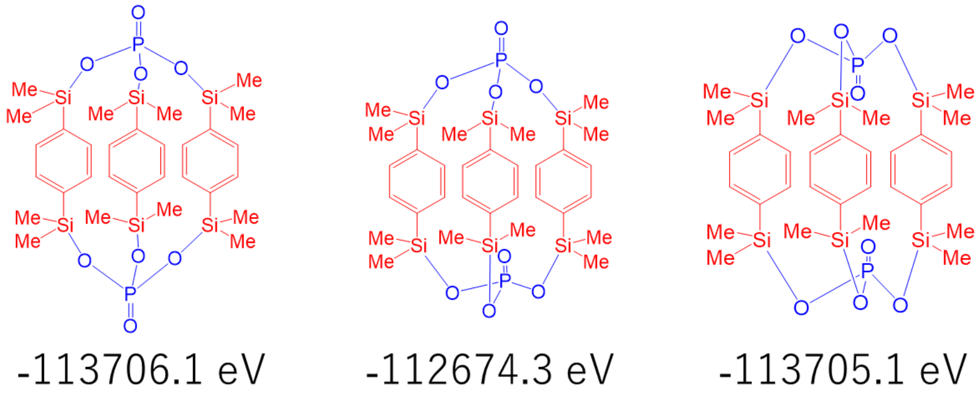

Figure S5. Possible molecular structures of a cage oligomer made from two phosphoric acid and three (C_6H_4)(Me_2SiCl)$_2$ molecules, which are calculated from DFT calculations.

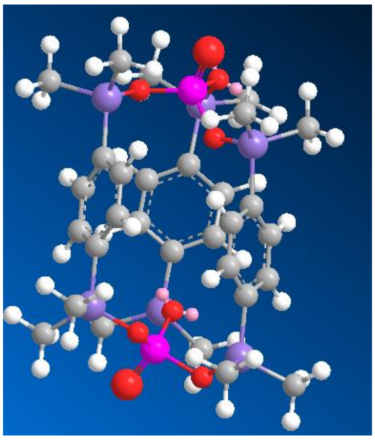

Figure S6. A stabilized molecular structure of cage oligomer made from two phosphoric acid and three $(C_6H_4)(Me_2SiCl)_2$ molecules.
